# Time-Cumulative Residual Cardiovascular Risk in Patients with Coronary Heart Disease and Diabetes: A 10-Year Follow-Up Study from a Large-Scale Population Cohort and an Independent Clinical Validation Cohort

**DOI:** 10.3390/jcdd13070306

**Published:** 2026-07-03

**Authors:** Zeping Li, Guangling Li, Yanan Wang, Jialin Zang, Luyun Wang, Jiangang Jiang

**Affiliations:** Department of Cardiology, Tongji Hospital, Tongji Medical College, Huazhong University of Science and Technology, Jiefang Avenue 1095, Wuhan 430030, China; d202482521@hust.edu.cn (Z.L.); d202382293@hust.edu.cn (G.L.); m202376681@hust.edu.cn (Y.W.); m202476601@hust.edu.cn (J.Z.)

**Keywords:** coronary heart disease, diabetes mellitus, residual risk, long-term prognosis, time-cumulative effect

## Abstract

Background: Patients with coronary heart disease (CHD) complicated by diabetes mellitus (DM) remain at substantial residual cardiovascular risk despite contemporary guideline-directed medical therapy. However, the long-term trajectory of this excess risk and its temporal pattern have not been fully clarified. Methods: This is a retrospective cohort study based on a large-scale public database and real-world clinical data. The primary cohort was derived from the UK Biobank (UKB), including 7491 CHD patients and 2322 CHD with DM patients; the validation cohort included 362 CHD patients from Tongji Hospital. Both cohorts were followed for up to 10 years, with major adverse cardiovascular events (MACE) as the primary endpoint. Propensity score matching (PSM) was employed to balance baseline confounders. Kaplan–Meier analysis combined with piecewise log-rank tests were used to assess cumulative risk differences at various follow-up time points. Multivariable Cox proportional hazards models were constructed to evaluate the independent impact of diabetes. Results: In the UKB cohort, CHD with DM patients exhibited significantly higher risks of MACE and cardiovascular death before matching. After 1:1 PSM, no significant difference in MACE risk was observed during the early follow-up period (1 year, *p* > 0.05). However, survival curves showed progressive divergence over time, with the risk difference reaching statistical significance at 10 years (*p* = 0.0004), demonstrating a pronounced time-cumulative effect. The Tongji validation cohort similarly confirmed that event-free survival was significantly lower in the CHD with DM group (*p* = 0.0028). Independent risk factor analysis using multivariable Cox regression showed that after adjusting for age, sex, smoking, and lipid parameters, diabetes remained an independent risk factor for long-term MACE (UKB cohort HR > 1; Tongji cohort HR = 1.86, 95% CI: 1.20–2.86, *p* = 0.005). Conclusions: Diabetes significantly increases the long-term residual cardiovascular risk in CHD patients. This excess risk is characterized by a clear time-cumulative effect: under modern guideline-directed medical therapy, early risk may be effectively buffered, but long-term adverse events remain markedly elevated. More proactive and intensified long-term intervention strategies are urgently needed for CHD patients with comorbid diabetes.

## 1. Introduction

Coronary heart disease (CHD) remains the leading cause of global disease burden and mortality [1]. In recent years, with the widespread implementation of percutaneous coronary intervention (PCI), coronary artery bypass grafting (CABG), and guideline-directed medical therapy (GDMT, including intensive lipid-lowering and dual antiplatelet therapies), acute-phase survival rates for CHD patients have significantly improved. However, mounting clinical evidence suggests that even after standardized revascularization and modern secondary prevention, a substantial proportion of CHD patients continue to experience recurrent cardiovascular events, such as myocardial infarction, stroke, and cardiovascular death, during long-term follow-up [2]. This suggests a vast, unmet need regarding the long-term residual cardiovascular risk within current intervention frameworks. Identifying the core drivers of this long-term residual risk has become a critical bottleneck to be addressed in cardiovascular secondary prevention.

Diabetes mellitus (DM) is a well-recognized extremely high-risk factor for cardiovascular disease and the most common metabolic comorbidity among CHD patients [3]. Chronic exposure to hyperglycemia leads to the extensive deposition of advanced glycation end products (AGEs), triggering profound oxidative stress and endothelial failure [4]. More importantly, diabetes-induced vascular injury exhibits a pronounced feature of metabolic memory, whereby long-term metabolic disturbances, through epigenetic mechanisms such as DNA methylation, histone modifications, and non-coding RNAs, maintain vascular-related cells in a persistently pathological activated state. This is manifested by abnormal proliferation/phenotypic switching of vascular smooth muscle cells, as well as proinflammatory and prothrombotic tendencies, thereby driving the persistent progression of diabetic vascular complications [5,6]. Such deep-seated pathological mechanisms imply that the detrimental impact of diabetes on CHD prognosis is not static but rather follows a cumulative trajectory that amplifies over time.

Despite numerous epidemiological studies exploring the link between DM and cardiovascular outcomes, several critical gaps remain. First, most research has focused on the primary risk of DM inducing CHD in general populations, leaving the “excess residual risk” contributed by DM in patients with established CHD insufficiently quantified [7]. Second, in the era of modern GDMT, intensified early interventions have fundamentally altered the natural history of cardiovascular disease. It remains unclear whether modern medical management merely masks or delays the excess risk brought by DM in the short term without abrogating its long-term pathological progression. Existing studies often have short follow-up durations (typically <3–5 years), failing to fully characterize the temporal trajectory of DM’s impact on CHD prognosis over a decade [8]. Furthermore, current findings are often limited to single healthcare systems or ethnic groups, lacking external validation across different regions and populations, which limits their global clinical applicability.

Addressing these evidence gaps, this study aimed to map the evolutionary trajectory of residual risk in CHD patients with comorbid diabetes during long-term follow-up. We leveraged the UK Biobank (UKB), a large-scale and representative prospective population cohort, and introduced an independent real-world clinical cohort from Tongji Hospital in China for cross-population validation. Through a 10-year follow-up and rigorous propensity score matching (PSM), we investigated whether diabetes independently drives major adverse cardiovascular events (MACE) after controlling for traditional baseline confounders. We specifically sought to unmask the “early-latent, late-explosive” time-cumulative characteristics of this residual risk under modern treatment paradigms. We anticipate that this cross-cohort evidence will provide a new perspective for precise risk stratification and the formulation of more proactive long-term secondary prevention strategies for CHD patients with comorbid diabetes.

## 2. Methods

### 2.1. Study Design and Population

This is a retrospective observational study based on a large-scale prospective population database and a real-world clinical cohort. The study population was derived from two independent cohorts: the UK Biobank (UKB) and a single-center inpatient cohort from Tongji Hospital.

**Figure 1:**
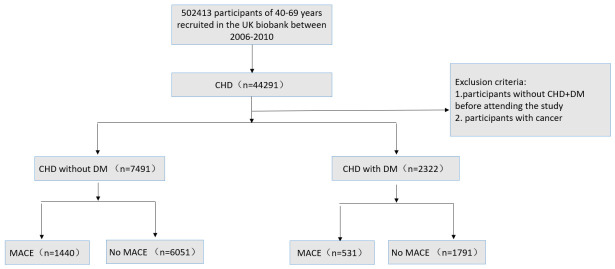
Flowchart of study participant selection and clinical outcomes in the UK Biobank cohort. A total of 502,413 participants aged 40–69 years recruited between 2006 and 2010 were initially screened. After excluding participants who did not meet the CHD or DM criteria before enrollment and those with a history of cancer, 44,291 patients with CHD were identified. The cohort was further stratified into two groups: CHD without DM (*n* = 7491) and CHD with DM (*n* = 2322). The occurrence of Major Adverse Cardiovascular Events (MACE) during the follow-up period is presented for both groups.

The UKB is a large-scale prospective cohort study that recruited 502,413 participants aged 40 to 69 years between 2006 and 2010. Based on baseline data and follow-up medical records, participants with a definitive diagnosis of coronary heart disease (CHD) were identified. These patients were subsequently stratified according to the presence of comorbid diabetes mellitus (DM) at baseline into a CHD without DM group (*n* = 7491) and a CHD with DM group (*n* = 2322). To ensure diagnostic accuracy and cohort homogeneity, patients with a history of malignancy at baseline and those failing to meet the relevant diagnostic criteria were excluded (Figure 1).

**Figure 2:**
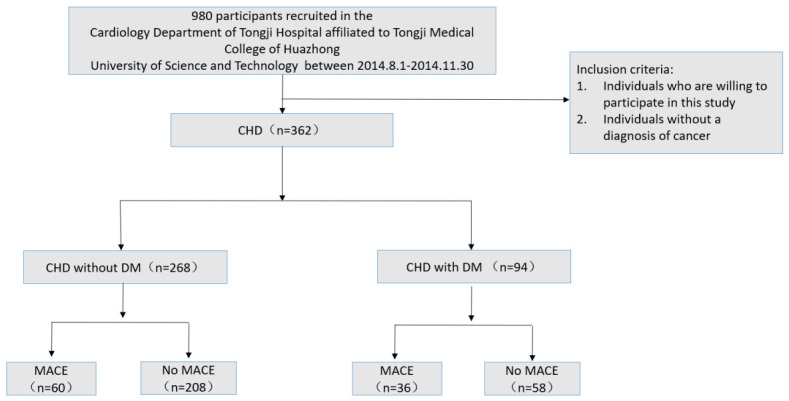
Flowchart of participant selection and clinical outcomes in Tongji Hospital, affiliated with Tongji Medical College of Huazhong University of Science and Technology validation cohort. A total of 980 participants were recruited from the Cardiology Department of Tongji Hospital, affiliated with Tongji Medical College of Huazhong.

Furthermore, to externally validate our findings, an independent clinical cohort comprising patients admitted to the Department of Cardiology at Tongji Hospital (Tongji Medical College, Huazhong University of Science and Technology) between August and December 2014 was included. A total of 362 patients with definitively diagnosed CHD were enrolled and similarly stratified based on the presence of comorbid DM (Figure 2).

Based on the inclusion criteria (willingness to participate and no prior diagnosis of cancer), 362 patients with CHD were included in the final validation analysis. The participants were categorized into the CHD without DM group (*n* = 268) and the CHD with DM group (*n* = 94). Clinical outcomes (MACE) were recorded for both groups during the 10-year follow-up.

### 2.2. Outcome Definitions

The primary endpoint of this study was major adverse cardiovascular events (MACE), defined as a composite of recurrent acute myocardial infarction (AMI), cardiovascular death, and stroke (including both ischemic and hemorrhagic stroke). Secondary endpoints included all-cause mortality, as well as AMI and stroke analyzed as individual components.

### 2.3. Follow-Up and Outcome Assessment

Long-term cardiovascular outcomes were longitudinally tracked using the UKB database linkages and the electronic medical records from Tongji Hospital. Cumulative event rates were estimated using the Kaplan–Meier method, and overall survival distributions between the two groups were compared using the log-rank test. To further evaluate the dynamic temporal evolution of risk differences between the two groups, piecewise survival analyses were conducted at prespecified landmark time points, specifically at 1 month (0.083 years), 6 months (0.5 years), 1 year, 3 years, 5 years, and 10 years.

### 2.4. Propensity Score Matching (pSM)

**Figure 3:**
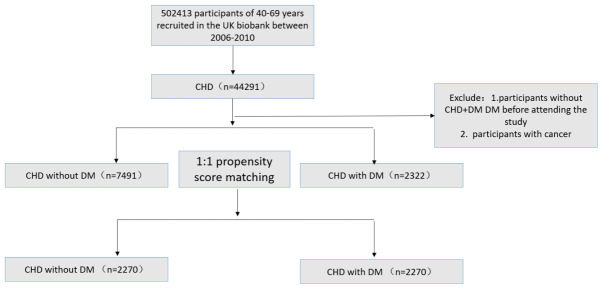
Flowchart of the 1:1 propensity score matching process in the UK Biobank cohort. The diagram illustrates the selection and matching process within the UK Biobank database. To minimize the influence of confounding factors, a 1:1 propensity score matching (PSM) was performed between patients with CHD without DM (n = 7491) and those with CHD and DM (n = 2322). This resulted in two well-balanced groups, each containing 2270 participants, for subsequent analysis of long-term residual cardiovascular risk.

Given the potential for significant baseline imbalances between the CHD and CHD with DM groups—such as higher body mass index (BMI), heavier metabolic burdens, and more complex pharmacological profiles in diabetic patients—a 1:1 propensity score matching (PSM) approach was implemented to minimize confounding bias. Propensity scores were calculated using a multivariable logistic regression model incorporating a comprehensive array of baseline covariates, including demographic characteristics, clinical indices, and relevant biochemical markers. The adequacy of covariate balance post-matching was assessed using the standardized mean difference (SMD), with an |SMD| < 0.1 considered indicative of optimal balance (Figure 3).

## 3. Statistical Analysis

Continuous variables are expressed as mean ± standard deviation (SD) for normally distributed data, or as median with interquartile range (IQR) for non-normally distributed data. Categorical variables are presented as frequencies and percentages (n, %). Baseline comparisons between groups were performed using the Student’s *t* test, Mann–Whitney U test, or Pearson’s chi-square test, as appropriate.

To evaluate the independent impact of diabetes on long-term adverse outcomes in CHD patients, Cox proportional hazards regression models were utilized to calculate hazard ratios (HRs) and 95% confidence intervals (CIs). In the multivariable models, adjustments were made for established traditional cardiovascular risk factors, including age, sex, BMI, and smoking status, to ascertain whether diabetes serves as an independent predictor of adverse cardiovascular events. All statistical analyses were two-sided, and a *p* value < 0.05 was considered statistically significant. R 4.4.2 were used for statistical analyses.

## 4. Results

### 4.1. Results of the UK Biobank (UKB) Cohort

#### 4.1.1. Study Population and Baseline Characteristics

Ultimately, 7491 eligible patients with coronary heart disease (CHD) alone and 2322 patients with CHD and comorbid diabetes mellitus (CHD with DM) were identified from the UK Biobank database. In the unmatched full cohort, 1440 incident major adverse cardiovascular events (MACE) occurred in the CHD group (6051 without MACE), whereas 531 MACE occurred in the CHD with DM group (1791 without MACE). This indicates that over a comparable follow-up period, both the absolute number and the proportion of cardiovascular events were higher in the CHD with DM group than in the isolated CHD group.

Baseline clinical characteristics revealed significant differences between the two groups across multiple risk factors and laboratory indices (Table 1). Overall, the CHD with DM group bore a heavier burden of metabolic risk factors. Several indicators of cardiac function and myocardial injury, including body mass index (BMI), cardiac troponin I (cTnI), and N-terminal pro-B-type natriuretic peptide (NT-proBNP), exhibited a more adverse distribution compared to the isolated CHD group, suggesting that CHD with DM patients were already at a higher global risk state at enrollment.

#### 4.1.2. Survival Analysis During Follow-Up

In the unmatched full cohort, cumulative event curves for CHD with DM patients progressively diverged from those of patients with CHD alone as follow-up extended.

**Figure 4:**
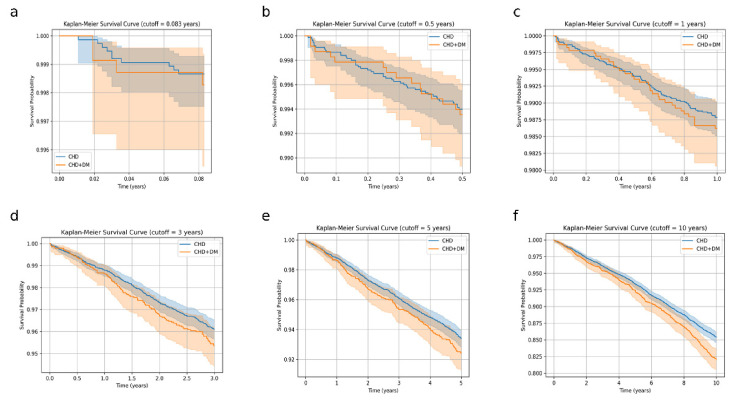
Time-segmented Kaplan–Meier survival curves comparing CHD and CHD with DM groups at different follow-up cutoffs. Kaplan–Meier survival analyses were performed separately using follow-up data truncated at 0.083 years (**a**), 0.5 years (**b**), 1 year (**c**), 3 years (**d**), 5 years (**e**), and 10 years (**f**). The blue line represents the CHD group and the orange line represents the CHD with DM group; shaded areas indicate 95% confidence intervals. Log-rank test results were as follows: 0.083 years, test statistic = 0.1871, *p* = 0.6653; 0.5 years, test statistic = 0.0593, *p* = 0.8076; 1 year, test statistic = 0.3804, *p* = 0.5374; 3 years, test statistic = 2.5755, *p* = 0.1085; 5 years, test statistic = 2.7269, *p* = 0.0987; and 10 years, test statistic = 14.9528, *p* = 0.0001. No significant difference in survival was observed between the two groups in the short- and mid-term analyses, whereas a significant separation emerged at the 10-year follow-up cutoff.

MACE Endpoint (Figure 4): Kaplan–Meier curves demonstrated no distinct difference between the two groups during the early follow-up phase (piecewise log-rank *p* > 0.05 at 1 month [0.083 years], 6 months [0.5 years], and 1 year). This suggests that conventional pharmacological therapies temporarily buffered the excess risk conferred by DM in the short term. However, starting from the third year of follow-up, the curves began to separate visibly, with the difference reaching high statistical significance by year 10 (*p* < 0.001). This indicates that the cumulative effect of DM on the residual risk of CHD unmasks over time, ultimately translating into a significant disparity in long-term prognosis.

Cardiovascular Death Endpoint (Figure S1): The divergence was even more pronounced. The cumulative incidence of cardiovascular death in the CHD with DM group trended upward early in the follow-up, with a clear separation of survival curves visible by year 1. Piecewise log-rank tests at 3, 5, and 10 years were all highly significant (overall log-rank *p* < 0.001), highlighting the profound detrimental impact of DM on long-term survival.

Acute Myocardial Infarction (AMI) and Stroke Endpoints (Figures S2 and S3): Similar trajectories were observed for these endpoints. Differences between the two groups were non-significant within the first year. However, from year 3 onward, the cumulative incidence in the CHD with DM group substantially exceeded that of the CHD group. Notably, the log-rank statistic for the stroke endpoint surpassed 40 at 10 years (*p* < 0.001), underscoring the formidable long-term driving force of diabetes on macrovascular and cerebrovascular events.

#### 4.1.3. Multivariable Cox Regression Analysis

**Figure 5:**
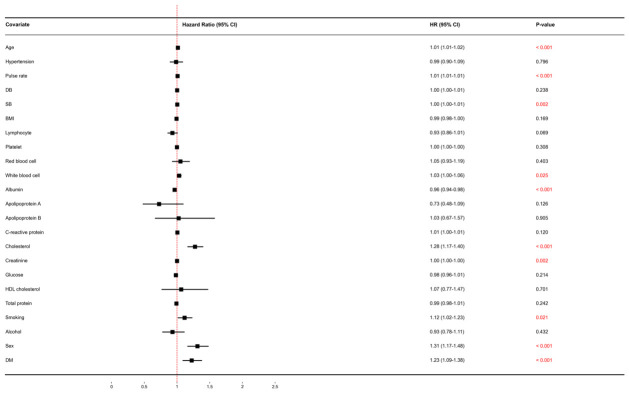
Forest plot of multivariate Cox proportional hazards regression analysis for MACE in the UK Biobank cohort. SB, systolic blood pressure; DB, diastolic blood pressure. *p*-values less than 0.05 are highlighted in red.

In the multivariable Cox regression analysis (Figure 5), when DM was incorporated as a covariate alongside adjustments for age, sex, smoking status, BMI, and other traditional risk factors, the risks of MACE, all-cause mortality, AMI, and stroke were significantly higher in the CHD with DM group than in the isolated CHD group. The hazard ratios (HRs) for all outcomes were >1, with 95% confidence intervals (CIs) not crossing 1. This confirms that even after controlling for common clinical risk factors, diabetes intrinsically remains a robust independent predictor of residual cardiovascular events in CHD patients.

The plot displays the hazard ratios (HRs) and 95% confidence intervals (CIs) for various demographic, clinical, and biochemical parameters. After adjusting for potential confounders including age, blood pressure, lipid profiles (Apolipoprotein A/B, Cholesterol), and lifestyle factors (smoking and alcohol status), diabetes mellitus (DM) remains a significant independent predictor of MACE (HR = 1.23, 95% CI: 1.09–1.38, *p* < 0.001). Red dots indicate significantly increased risk, while the green dot indicates a significant protective factor (Albumin).

Subsequent multivariable Cox regression utilizing cardiovascular death (CV death) as the endpoint (Figure S4) revealed that diabetes is an independent risk factor for cardiovascular mortality (HR = 1.54, 95% CI: 1.26–1.86, *p* < 0.001). Additionally, advanced age, hypertension, elevated pulse rate, higher systolic blood pressure, BMI, white blood cell count, smoking, and male sex were significantly associated with an increased risk of CV death, whereas lymphocyte count, red blood cell count, and diastolic blood pressure correlated with reduced risk.

Results for the AMI endpoint (Figure S5) were highly consistent. Diabetes remained an independent predictor of AMI (HR = 1.54, 95% CI: 1.26–1.86, *p* < 0.001).

Similarly, multivariable Cox regression for the stroke endpoint (Figure S6) demonstrated that diabetes independently increased stroke risk (HR = 1.44, 95% CI: 1.26–1.65, *p* < 0.001).

Overall, diabetes consistently emerged as an independent risk factor across diverse endpoints in the multivariable models, affirming its close association not only with the overarching MACE risk but also with specific adverse outcomes including CV death, AMI, and stroke.

#### 4.1.4. Propensity Score Matching and Post-Matching Baseline Characteristics

To rigorously mitigate the influence of baseline confounding, a 1:1 propensity score matching (PSM) approach was executed. After balancing demographic features, traditional risk factors, and key biochemical parameters, a matched cohort of 2270 pairs of CHD and CHD with DM patients was generated. Post-matching, the standardized mean differences (SMDs) for the vast majority of covariates were <0.1, indicating optimal control of baseline discrepancies between the groups (Table 2).

#### 4.1.5. Survival and Cox Regression Analysis in the Matched Cohort

**Figure 6:**
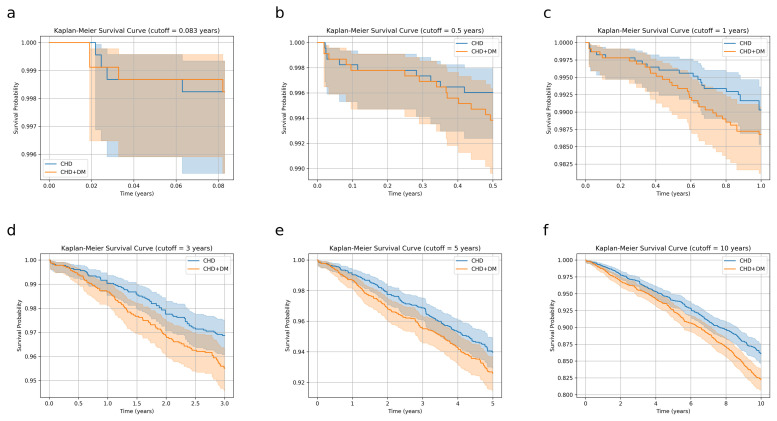
Kaplan–Meier survival curves comparing CHD and CHD with DM groups at different follow-up cutoffs (Matched). Kaplan–Meier survival analyses were performed for follow-up data truncated at 0.083 years (**a**), 0.5 years (**b**), 1 year (**c**), 3 years (**d**), 5 years (**e**), and 10 years (**f**). The blue line represents the CHD group, and the orange line represents the CHD with DM group. Shaded areas indicate the 95% confidence intervals. The results of the log-rank tests were as follows: 0.083 years: *p* = 0.9999; 0.5 years: test statistic = 1.0889, *p* = 0.2967; 1 year: *p* = 0.7625; 3 years: *p* = 0.0634; 5 years: *p* = 0.0634; 10 years: *p* = 0.0004.

Kaplan–Meier curves for MACE were reconstructed within the matched cohort (Figure 6). During the first year of follow-up, the cumulative event curves for both groups virtually overlapped (piecewise log-rank *p* > 0.25), demonstrating that upon adequate equalization of baseline variances, the short-term MACE risk is comparable between CHD with DM and CHD patients. Nevertheless, the curves began to diverge between 3 and 5 years, culminating in a statistically significant difference at 10 years (log-rank χ^2^ = 12.73, *p* = 0.0004). This aligns perfectly with the full-cohort analysis, firmly validating the pronounced time-cumulative nature of diabetic detriment in CHD patients.

**Figure 7:**
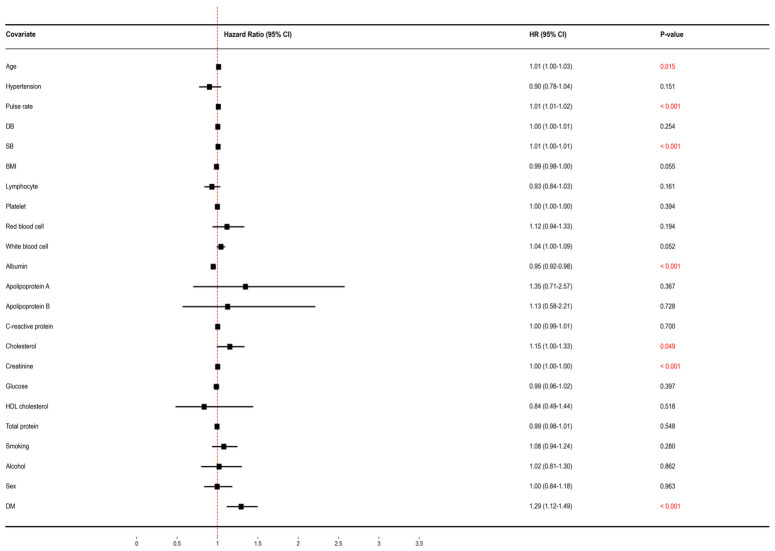
Hazard ratios from Cox regression analysis for MACE (Matched). This figure displays the results of a multivariable Cox regression analysis after matching, showing the associations between various factors and the risk of major adverse cardiovascular events (MACE). Each factor is listed along the vertical axis, with its corresponding hazard ratio (HR) and 95% confidence interval (CI) shown on the right. *p*-values less than 0.05 are highlighted in red.

Multivariable Cox regression in the matched cohort (Figure 7) corroborated these findings. Adjusting for traditional risk factors, the risks of MACE, mortality, AMI, and stroke remained significantly elevated in the CHD with DM group, unequivocally establishing diabetes as a crucial independent predictor of residual cardiovascular events.

### 4.2. Results of the Tongji Hospital Validation Cohort

#### 4.2.1. Baseline Characteristics

The independent Tongji Hospital cohort enrolled 362 CHD patients, comprising 94 with comorbid diabetes (CHD with DM) and 268 without diabetes (Non-DM) (Table 3). Age (59.28 ± 9.87 vs. 59.29 ± 10.27 years, *p* = 0.991) and sex distributions were comparable between the two groups. The prevalence of hypertension was significantly higher in the diabetic group (71.3% vs. 51.9%, *p* = 0.002), whereas the smoking rate was lower (34.0% vs. 46.6%, *p* = 0.046). Laboratory assays showed significantly elevated fasting blood glucose (7.69 vs. 5.52 mmol/L, *p* < 0.001), glycated hemoglobin (HbA1c, 6.80% vs. 5.80%, *p* < 0.001), and triglycerides (1.61 vs. 1.40 mmol/L, *p* = 0.005) in the diabetic group. No significant differences were detected in prior medical history (stroke, peripheral artery disease, arrhythmias, previous MI or CABG), ACS presentation type, Killip class, or the proportion of triple-vessel disease. Furthermore, the prescription rates for aspirin and statins were 100% in both groups, with no significant discrepancies in the utilization of other secondary prevention medications.

#### 4.2.2. Long-Term Follow-Up and Survival Analysis

**Figure 8:**
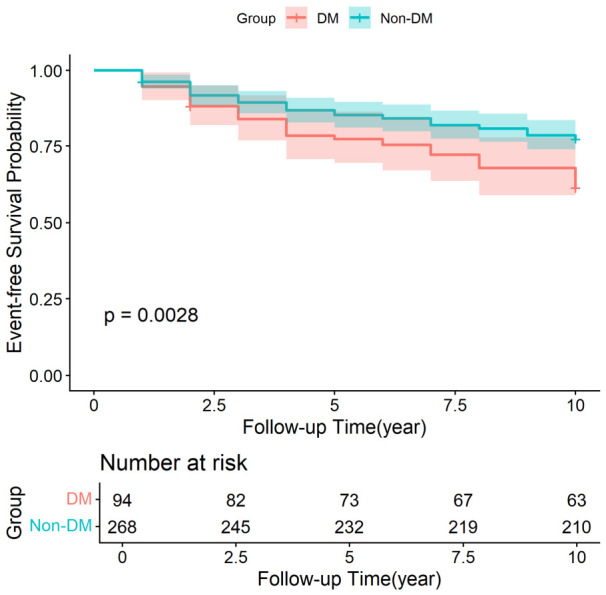
Kaplan–Meier curves for event-free survival in CHD patients with and without diabetes mellitus.

This cohort underwent extensive long-term tracking for up to 10 years, with only 6 patients lost to follow-up. Kaplan–Meier survival analysis (Figure 8) revealed that the event-free survival rate in the CHD with DM group was significantly lower than that in the non-diabetic group. The two survival curves began to separate early in the follow-up period, and the gap widened relentlessly over time. The log-rank test confirmed a statistically significant difference in event-free survival between the cohorts (*p* = 0.0028). This signifies that in real-world clinical practice, CHD patients with comorbid diabetes confront a substantially heightened long-term residual cardiovascular risk.

#### 4.2.3. Multivariable Cox Proportional Hazards Model Analysis

To thoroughly evaluate the impact of diabetes as a risk factor, three progressively adjusted Cox regression models were constructed (Table 4) to analyze its effect on MACE, cardiovascular death, AMI, and stroke:

MACE: In the unadjusted model (Model 1) and the age- and sex-adjusted model (Model 2), diabetes was significantly associated with MACE risk (*p* = 0.003). In the fully adjusted model (Model 3, further adjusting for hypertension, smoking, etc.), the association was slightly attenuated but remained robustly significant (HR = 1.816, 95% CI: 1.168–2.824, *p* = 0.008).

Cardiovascular Death and Stroke: Diabetes significantly increased the risk of both CV death and stroke in Models 1 and 2 (*p* < 0.05). However, under the most stringent covariate adjustments (Model 3), the association with CV death lost statistical significance (*p* = 0.164), while the risk for stroke became borderline significant (*p* = 0.065).

AMI: No statistically significant association between diabetes and the incidence of AMI was observed across any of the three models (all *p* > 0.3).

An overarching, fully adjusted multivariable Cox proportional hazards model (incorporating age, triple-vessel disease, sex, triglycerides, eGFR, and albumin as confounders; detailed in the Figure S7) decisively confirmed that diabetes (DM) remains an independent risk factor for adverse cardiovascular events (HR = 1.86, 95% CI: 1.20–2.86, *p* = 0.005). This indicates that CHD patients with comorbid diabetes are 1.86 times more likely to experience adverse events compared to their non-diabetic counterparts.

## 5. Discussion

Based on a large-scale prospective population cohort (UK Biobank) and an independent real-world clinical cohort (Tongji Hospital), this study systematically investigated the impact of diabetes on the long-term residual cardiovascular risk in patients with coronary heart disease (CHD). Our core finding is that in the era of modern standardized secondary prevention, diabetes remains an independent risk factor driving long-term major adverse cardiovascular events (MACE) in CHD patients. Crucially, this residual risk exhibits a unique “biphasic risk trajectory” and a pronounced time-cumulative effect. Over a 10-year follow-up, early risk differences were negligible; however, as time progressed, the excess risk conferred by DM gradually unmasked and amplified exponentially. This consistent finding across diverse cohorts and populations provides critical new insights into the long-term clinical management of CHD patients with comorbid diabetes [9].

The most significant academic contribution of this study lies in revealing the “time-cumulative effect” of diabetes on CHD residual risk. After rigorous propensity score matching (PSM) to adequately balance baseline confounders, the incidence curves of MACE for both groups nearly overlapped during the first year of follow-up. This phenomenon suggests that guideline-directed medical therapy (GDMT, including intensive statin therapy, dual antiplatelet therapy, and revascularization) profoundly buffers the acute prothrombotic and proinflammatory effects of diabetes in the short term. However, GDMT appears to merely “delay” rather than “abrogate” the deep-seated detriments of diabetes. After 3 to 5 years of follow-up, the curves progressively diverged, reaching high statistical significance at 10 years. We postulate that this late-stage resurgence of residual risk profoundly reflects the “metabolic memory” [10] characteristic of diabetic vasculopathy.

Mechanistically, diabetes is not merely an anomaly of glycemic indices but a persistent state of systemic metabolic toxicity. Chronic hyperglycemia and insulin resistance activate the polyol pathway, promote the extensive deposition of advanced glycation end products (AGEs), and upregulate the protein kinase C (PKC) signaling pathway, culminating in profound oxidative stress and endothelial exhaustion [11]. Even if perfect anatomical restoration (e.g., via PCI) is achieved during the acute phase of CHD, diabetes-induced phenotypic switching of smooth muscle cells, chronic low-grade inflammation (enhanced intra-plaque macrophage infiltration), and persistent microcirculatory dysfunction probably continue to drive slow, irreversible epigenetic alterations within the myocardium and vascular walls. It is the long-term interplay and accumulation of these multiple pathological pathways that ultimately breach the protective barrier of conventional pharmacotherapy, translating into a high incidence of long-term MACE.

Notably, our study observed heterogeneity in the impact of diabetes on different cardiovascular endpoints. For overall MACE and stroke, diabetes exerted a robust and significant risk-amplifying effect. Conversely, in the Tongji single-center cohort, the statistical association of diabetes with acute myocardial infarction (AMI) and cardiovascular death was relatively attenuated. We attribute this to two factors: first, the limited number of events for specific single endpoints in the validation cohort may have reduced statistical power; second, this likely reflects the paradigm of modern cardiovascular interventions. Current high-intensity antithrombotic and lipid-lowering therapies are highly efficacious in preventing acute large-vessel occlusions (such as AMI) but remain inadequate against diabetes-specific microangiopathy, diffuse vascular stiffening, and extensive cerebrovascular damage (such as stroke) [12]. This implies that the long-term cerebral vascular damage inflicted by diabetes may be more insidious and resistant to conventional cardiovascular drugs.

In recent years, the early control of cardiovascular risk factors has gained significant attention. For instance, a recent study highlighted that controlling hypertension before 55 to 60 years of age, along with smoking cessation, is associated with the greatest increase in life-years free of cardiovascular disease [13]. However, unlike hypertension and smoking, which often manifest as apparent risks early in life, the cardiovascular burden associated with diabetes typically becomes particularly prominent in later decades of life (e.g., the seventh and eighth decades in elderly populations). Given the ongoing pandemic of type 2 diabetes, neglecting diabetes management in the elderly population can result in exceptionally high morbidity and mortality [14]. Therefore, our 10-year follow-up study of patients with concomitant coronary heart disease and diabetes holds crucial clinical significance for evaluating and managing this late-stage, time-cumulative residual risk.

This study has several limitations. First, despite the rigorous multivariable adjustment and PSM employed to maximize baseline comparability, as a retrospective observational study, residual or unmeasured confounding (e.g., long-term medication adherence, specific anatomical complexity of coronary lesions, and lifestyle/dietary interventions) cannot be entirely ruled out. Second, constrained by the inherent nature of the databases, we lacked continuous glucose monitoring data (e.g., longitudinal trajectories of HbA1c) throughout the follow-up period, precluding the exact quantification of the contribution of long-term glycemic variability or cumulative metabolic exposure to residual risk. Third, given the extensive 10-year follow-up span, we could not comprehensively track and dynamically adjust for the initiation of novel targeted hypoglycemic agents with proven cardiovascular benefits (e.g., SGLT2 inhibitors or GLP-1 receptor agonists) in the mid-to-late phases of the historical cohorts. Finally, this study did not explicitly stratify type 1 and type 2 diabetes; although the vast majority of patients with comorbid CHD have type 2 diabetes, the absence of this distinction might slightly obscure the subtle differences in cardiovascular risk trajectories driven by distinct pathophysiological mechanisms (i.e., absolute insulin deficiency versus severe insulin resistance). Another limitation of this study is the lack of baseline data on triglycerides (TG) and specific cholesterol subfractions (such as LDL-C), which reflect remnant cholesterol and are likely important factors in residual risk among these patients. These remaining questions warrant further elucidation in future large-scale prospective randomized controlled trials (RCTs) or disease-specific cohorts with more granular, multi-dimensional clinical records.

The findings of this study harbor compelling clinical translational implications. They issue a clear caveat: the current CHD management paradigm, centered on “anatomical revascularization” and “traditional risk factor control (blood pressure and lipids),” is insufficient to address the lifelong cardiovascular risk of patients with comorbid diabetes. Clinical practice must undergo a paradigm shift from “post-event intervention” to “early and proactive metabolic modulation.” Building upon traditional secondary prevention, novel targeted metabolic therapies with proven cardiovascular benefits (such as SGLT2 inhibitors and GLP-1 receptor agonists) should be integrated earlier and more broadly into the management pathways of CHD with DM patients [15]. This multi-dimensional approach—targeting energy metabolism, systemic inflammation, and endothelial protection—is urgently needed to fundamentally attenuate this time-dependent escalation of residual risk.

## 6. Conclusions

Through cross-validation between a large-scale prospective population cohort and an independent real-world clinical cohort, this study firmly establishes diabetes as a core determinant driving the long-term residual cardiovascular risk in CHD patients. Our findings innovatively unveil the “time-cumulative effect” of this excess risk: even under modern standardized secondary prevention, where early risk may be transiently masked, CHD patients with comorbid diabetes still face a significantly escalating risk of cardiovascular death, stroke, and overall MACE during long-term follow-up (5–10 years). This consistent cross-cohort evidence strongly advocates that for this extremely high-risk population, the current intervention paradigm must urgently transition toward an “early, proactive, and lifelong comprehensive metabolic intervention” to bridge the profound gaps of conventional cardiovascular secondary prevention in mitigating deep-seated metabolic damage.

## Figures and Tables

**Table 1:** Baseline characteristics of the UK Biobank cohort.

Variable	Overall	CHD Without DM	CHD with DM	*p* Value
	(*n* = 9813)	(*n* = 7491)	(*n* = 2322)	
Age, years	60.51 (6.57)	60.51 (6.58)	60.51 (6.55)	0.986
Male, n (%)	6358 (64.79)	4804 (64.13)	1554 (66.93)	0.015
Hypertension	5245 (53.45)	3664 (48.91)	1581 (68.09)	<0.001
Pulse rate	66.99 (12.94)	65.52 (12.34)	71.71 (13.68)	<0.001
DBP, mmHg	80.29 (11.08)	80.39 (11.07)	79.97 (11.12)	0.124
SBP, mmHg	140.85 (19.98)	140.56 (20.02)	141.76 (19.81)	0.015
BMI, kg/m^2^	29.17 (5.12)	28.39 (4.63)	31.72 (5.78)	<0.001
Smoking	5519 (56.24)	4147 (55.36)	1372 (59.09)	0.002
Drinking	9098 (92.71)	7044 (94.03)	2054 (88.46)	<0.001
Lymphocyte, ×10^9^/L	2.00 (0.84)	1.97 (0.64)	2.11 (1.29)	<0.001
Platelet, ×10^9^/L	242.90 (62.71)	242.08 (60.92)	245.59 (68.21)	0.032
Red blood cell, ×10^12^/L	4.57 (0.43)	4.56 (0.42)	4.58 (0.46)	0.216
White blood cell, ×10^9^/L	7.10 [6.04, 8.40]	6.98 [5.93, 8.25]	7.60 [6.45, 8.98]	<0.001
Albumin, g/L	44.98 (2.77)	45.05 (2.68)	44.76 (3.03)	<0.001
Apolipoprotein A, g/L	1.42 (0.25)	1.45 (0.25)	1.35 (0.24)	<0.001
Apolipoprotein B, g/L	0.91 (0.24)	0.92 (0.24)	0.88 (0.24)	<0.001
C-reactive protein, mg/L	1.58 [0.76, 3.31]	1.43 [0.70, 3.00]	2.17 [1.05, 4.53]	<0.001
Cholesterol, mmol/L	4.86 (1.21)	4.94 (1.20)	4.58 (1.21)	<0.001
Creatinine, μmol/L	79.58 (33.19)	78.41 (25.88)	83.43 (50.06)	<0.001
Glucose, mmol/L	5.07 [4.68, 5.60]	4.96 [4.63, 5.34]	6.02 [5.08, 7.93]	<0.001
HDL cholesterol, mmol/L	1.26 (0.34)	1.29 (0.34)	1.14 (0.30)	<0.001
Total protein, g/L	72.42 (4.28)	72.31 (4.18)	72.79 (4.60)	<0.001

Values are presented as mean (SD), median [IQR], or n (%), as appropriate. *p* values were calculated using Student’s *t* test or Mann–Whitney U test for continuous variables and the chi-square test for categorical variables. Overall, CHD without DM and CHD with DM groups are shown. DBP, diastolic blood pressure; SBP, systolic blood pressure; BMI, body mass index; HDL, high-density lipoprotein.

**Table 2:** Baseline characteristics of the UK Biobank cohort after propensity score matching (PSM).

Variable	Overall	CHD Without DM	CHD with DM	*p* Value
	(*n* = 4540)	(*n* = 2270)	(*n* = 2270)	
Age, years	60.51 (6.57)	60.45 (6.63)	60.57 (6.51)	0.542
Male, n (%)	3056 (67.31)	1544 (68.02)	1512 (66.61)	0.327
Hypertension	3120 (68.72)	1567 (69.03)	1553 (68.41)	0.677
Pulse rate	68.90 (13.47)	66.17 (12.67)	71.61 (13.68)	<0.001
DBP, mmHg	81.12 (11.19)	82.26 (11.11)	79.99 (11.16)	<0.001
SBP, mmHg	142.66 (19.93)	143.62 (19.97)	141.71 (19.85)	0.002
BMI, kg/m^2^	31.69 (5.82)	31.67 (5.86)	31.72 (5.78)	0.775
Smoking	2719 (59.89)	1374 (60.53)	1345 (59.25)	0.397
Drinking	4143 (91.26)	2131 (93.88)	2012 (88.63)	<0.001
Lymphocyte, ×10^9^/L	2.06 (1.03)	2.02 (0.65)	2.11 (1.30)	0.002
Platelet, ×10^9^/L	243.87 (65.94)	242.36 (64.39)	245.42 (67.46)	0.129
RBC, ×10^12^/L	4.58 (0.44)	4.59 (0.42)	4.58 (0.45)	0.306
WBC, ×10^9^/L	7.40 [6.30, 8.70]	7.16 [6.19, 8.46]	7.60 [6.43, 8.96]	<0.001
Albumin, g/L	44.82 (2.86)	44.85 (2.69)	44.79 (3.02)	0.486
Apo A, g/L	1.38 (0.24)	1.41 (0.24)	1.35 (0.24)	<0.001
Apo B, g/L	0.90 (0.24)	0.91 (0.23)	0.88 (0.24)	<0.001
CRP, mg/L	1.99 [0.98, 4.18]	1.90 [0.94,3.95]	2.13 [1.04, 4.44]	0.002
Cholesterol, mmol/L	4.71 (1.18)	4.84 (1.14)	4.58 (1.21)	<0.001
Creatinine, μmol/L	81.36 (34.90)	79.90 (19.66)	82.85 (45.45)	0.006
Glucose, mmol/L	5.27 [4.80, 6.31]	5.04 [4.68, 5.39]	6.00 [5.08, 7.90]	<0.001
HDL, mmol/L	1.19 (0.31)	1.23 (0.30)	1.14 (0.30)	<0.001
Total protein, g/L	72.58 (4.38)	72.38 (4.16)	72.79 (4.59)	0.004

Values are presented as mean (SD), median [IQR], or n (%), as appropriate. *p* values were calculated using Student’s *t* test or Mann–Whitney U test for continuous variables and the chi-square test for categorical variables. Overall, CHD without DM, and CHD with DM groups are shown. DBP, diastolic blood pressure; SBP, systolic blood pressure; BMI, body mass index; RBC, red blood cell count; WBC, white blood cell count; Apo A, apolipoprotein A; Apo B, apolipoprotein B; CRP, C-reactive protein; HDL, high-density lipoprotein.

**Table 3:** Baseline characteristics of the study population from Tongji Hospital.

Characteristics	Overall	DM	Non-DM	*p*
	n = 362	n = 94	n = 268	
Age, years	59.29 (10.15)	59.28 (9.87)	59.29 (10.27)	0.991
Male, n (%)	257 (71.0)	60 (63.8)	197 (73.5)	0.1
Risk factors				
Smoking	157 (43.4)	32 (34.0)	125 (46.6)	0.046
Hypertension	206 (56.9)	67 (71.3)	139 (51.9)	0.002
Medical history				
Previous Stroke	18 (5.0)	2 (2.1)	16 (6.0)	0.231
Previous PAD	42 (11.6)	11 (11.7)	31 (11.6)	1
Arrhythmia	23 (6.4)	6 (6.4)	17 (6.3)	1
COPD	9 (2.5)	0 (0.0)	9 (3.4)	0.157
Previous MI or CABG	18 (5)	4 (4.3)	14(5.3)	0.703
Glucose, mmol/L	5.75 [5.11, 7.18]	7.69 [6.17, 10.84]	5.52 [5.00, 6.28]	<0.001
HbA1c, %	5.90 [5.60, 6.40]	6.80 [6.30, 7.50]	5.80 [5.60, 6.00]	<0.001
Albumin, g/L	41.25 [38.70, 43.10]	41.95 [39.62, 43.77]	41.10 [38.38, 43.00]	0.056
CRP, mg/L	2.50 [0.80, 5.00]	2.80 [1.30, 5.00]	2.32 [0.67, 5.05]	0.333
Creatinine, μmol/L	74.00 [65.00, 85.00]	74.00 [62.00, 88.75]	74.00 [65.00, 83.00]	0.861
EGFR	89.10 [72.10, 100.50]	87.35 [67.80, 98.30]	89.20 [72.25, 101.20]	0.594
LDL-C, mmol/L	2.39 [1.90, 3.10]	2.42 [1.88, 3.18]	2.39 [1.93, 2.97]	0.489
TG, mmol/L	1.40 [0.99, 2.08]	1.61 [1.12, 2.43]	1.40 [0.97, 1.86]	0.005
HDL-C, mmol/L	0.95 [0.83, 1.08]	0.91 [0.80, 1.05]	0.95 [0.83, 1.10]	0.148
TC, mmol/L	3.99 [3.36, 4.70]	4.12 [3.45, 4.84]	3.99 [3.35, 4.64]	0.336
Three-Vessel Disease	4 (1.1)	1 (1.1)	3 (1.1)	1
Diagnosis of ACS				
ST-elevation MI	87 (24.0)	25 (26.6)	62 (23.1)	0.238
Non-ST-elevation MI	61 (16.9)	20 (21.3)	41 (15.3)	
Unstable angina	214 (59.1)	49 (52.1)	165 (61.6)	
Killip class > 2	11 (3.0)	3 (3.2)	8 (3.0)	1
Medications				
ACE inhibitors	234 (64.6)	65 (69.1)	169 (63.1)	0.349
Beta-blockers	273 (75.4)	78 (83.0)	195 (72.8)	0.066
Organic nitrate	103 (28.5)	32 (34.0)	71 (26.5)	0.207
Diuretics	16 (4.4)	7 (7.4)	9 (3.4)	0.171
Aspirin	362	94 (100)	268 (100)	1
Statins	362	94 (100)	268 (100)	1

Values are presented as mean (SD), median [IQR], or n (%), as appropriate. MI, myocardial infarction; CABG, coronary artery bypass graft; PAD, peripheral artery disease; COPD, chronic obstructive pulmonary disease; TC, total cholesterol; TG, triglycerides; HDL-C, high-density lipoprotein cholesterol; LDL-C, low-density lipoprotein cholesterol; HbA1c, glycated hemoglobin; ACS, acute coronary artery syndrome; ACE, angiotensin-converting enzyme. With 1829 missing values.

**Table 4:** HRs (95% CIs) for cardiovascular events according to DM.

Outcome	Model 1		Model 2		Model 3	
	HR (95%CI)	*p*	HR (95%CI)	*p*	HR (95%CI)	*p*
MACE	1.864 (1.233, 2.818)	0.003	1.900 (1.250, 2.888)	0.003	1.816 (1.168, 2.824)	0.008
CV death	3.659 (0.983, 13.626)	0.053	4.211 (1.113, 15.927)	0.034	2.884 (0.648, 12.832)	0.164
AMI	1.272 (0.743, 2.179)	0.38	1.308 (0.761, 2.248)	0.332	1.343 (0.758, 2.378)	0.312
Stroke	2.659 (1.173, 6.027)	0.019	2.439 (1.068, 5.571)	0.034	2.297 (0.950, 5.556)	0.065

Model 1: Unadjusted. Model 2: Adjusted for age and sex. Model 3 for MACE endpoint: Additionally adjusted for hypertension, smoking, Killip class, three-vessel disease, TC, HDL-C, TG, albumin, LDL-C, aspirin, statin, ACEI, and β-blocker. Model 3 for secondary endpoints: additionally adjusted for hypertension and smoking.

## Data Availability

The data presented in this study are available on request from the corresponding author due to ethical restrictions. UK Biobank data were used under license and are available through application to the UK Biobank Access Management System (https://www.ukbiobank.ac.uk/, accessed on 1 February 2026).

## Supplementary Materials

The following supporting information can be downloaded at: https://www.mdpi.com/article/10.3390/jcdd13070306/s1, Figure S1. Time-segmented Kaplan–Meier survival curves for cardiovascular death in patients with CHD and CHD+DM. Figure S2. Time-segmented Kaplan–Meier curves for acute myocardial infarction in the CHD and CHD+DM groups. Figure S3. Time-segmented Kaplan–Meier curves for stroke in the CHD and CHD+DM groups. Figure S4. Forest plot of multivariable Cox regression analysis for cardiovascular death in patients with CHD. Figure S5. Forest plot of multivariable Cox regression analysis for acute myocardial infarction in patients with CHD. Figure S6. Forest plot of multivariable Cox regression analysis for stroke in patients with CHD. Figure S7. Forest plot of multivariable Cox regression analysis for MACE in patients of Tongji Hospital cohort.

## Abbreviations

CHD	coronary heart disease
DM	diabetes mellitus
MACE	major adverse cardiovascular events
AMI	acute myocardial infarction;
CV	cardiovascular
ACS	acute coronary syndrome
PCI	percutaneous coronary intervention
CABG	coronary artery bypass grafting
PSM	propensity score matching
HR	hazard ratio
CI	confidence interval
IQR	interquartile range
BMI	body mass index
SBP	systolic blood pressure
DBP	diastolic blood pressure
CRP	C-reactive protein
HbA1c	glycated hemoglobin
eGFR	estimated glomerular filtration rate
TG	triglyceride
TC	total cholesterol
ACE	angiotensin-converting enzyme
