# Supplementary material for: Time-Cumulative Residual Cardiovascular Risk in Patients with Coronary Heart Disease and Diabetes: A 10-Year Follow-Up Study from a Large-Scale Population Cohort and an Independent Clinical Validation Cohort
Source: J Cardiovasc Dev Dis. 2026 Jul 3;13(7):306. doi: 10.3390/jcdd13070306 (PMC13409900; doi:10.3390/jcdd13070306)

Figure S1. Time-segmented Kaplan–Meier survival curves for cardiovascular death in patients with CHD and CHD+DM.

Kaplan–Meier curves are shown for different follow-up intervals: (a) 0.083 years, (b) 0.5 years, (c) 1 year, (d) 3 years, (e) 5 years, and (f) 10 years. The blue curve represents patients with CHD alone, and the orange curve represents patients with CHD combined with diabetes mellitus (CHD+DM). Shaded areas indicate 95% confidence intervals.

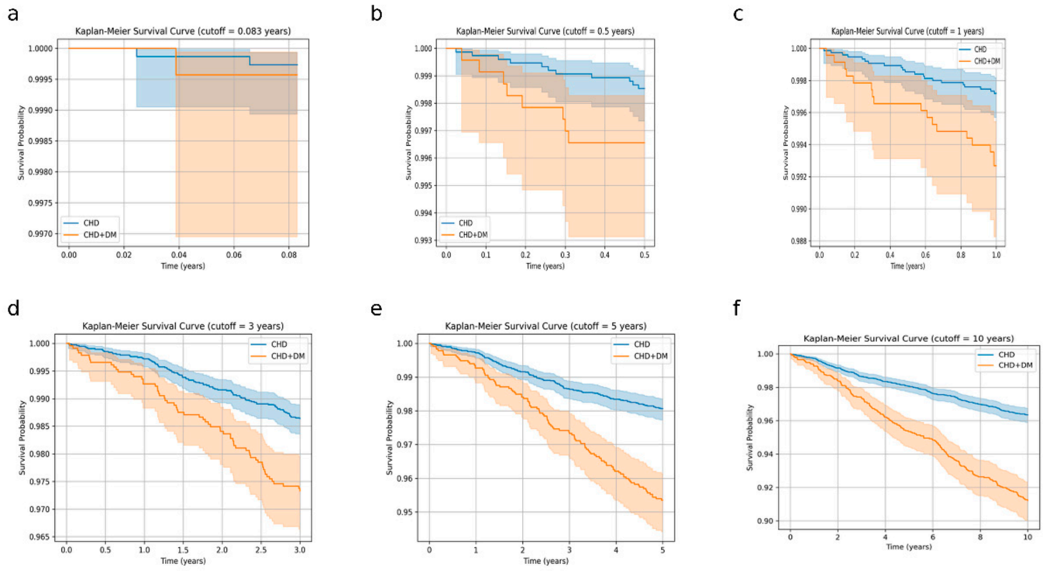

Figure S2. Time-segmented Kaplan–Meier curves for acute myocardial infarction in the CHD and CHD+DM groups.

(a) 0.083 years; (b) 0.5 years; (c) 1 year; (d) 3 years; (e) 5 years; and (f) 10 years. Blue indicates CHD, and orange indicates CHD+DM. Shaded areas represent 95% confidence intervals.

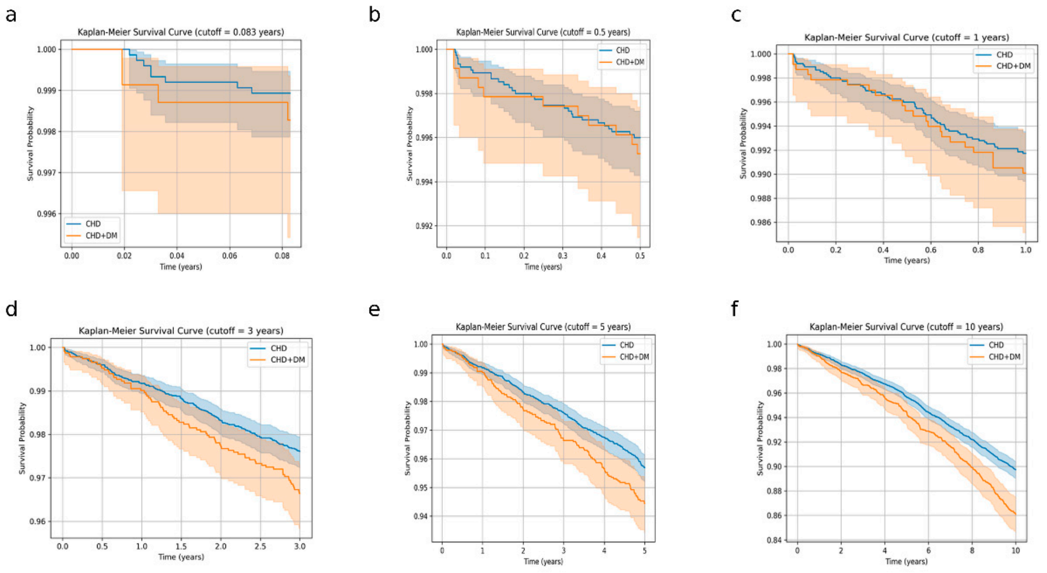

Figure S3. Time-segmented Kaplan–Meier curves for stroke in the CHD and CHD+DM groups.

(a) 0.083 years; (b) 0.5 years; (c) 1 year; (d) 3 years; (e) 5 years; and (f) 10 years. Blue indicates CHD, and orange indicates CHD+DM. Shaded areas represent 95% confidence intervals.

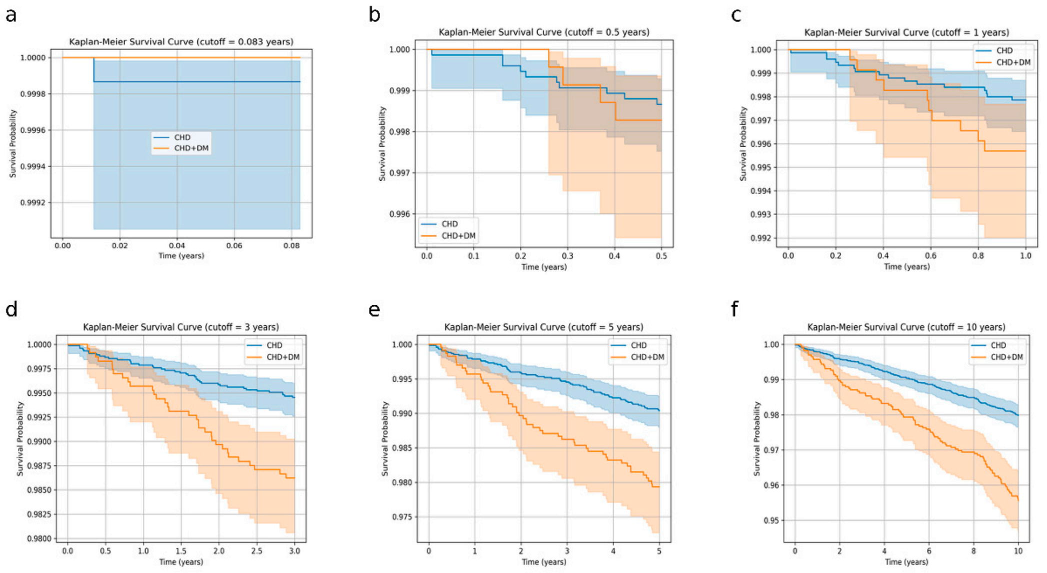

Figure S4. Forest plot of multivariable Cox regression analysis for cardiovascular death in patients with CHD.

Hazard ratios (HRs) with 95% confidence intervals (CIs) and corresponding P values are shown for each covariate included in the model. The red dashed vertical line indicates the reference value of HR = 1.0.

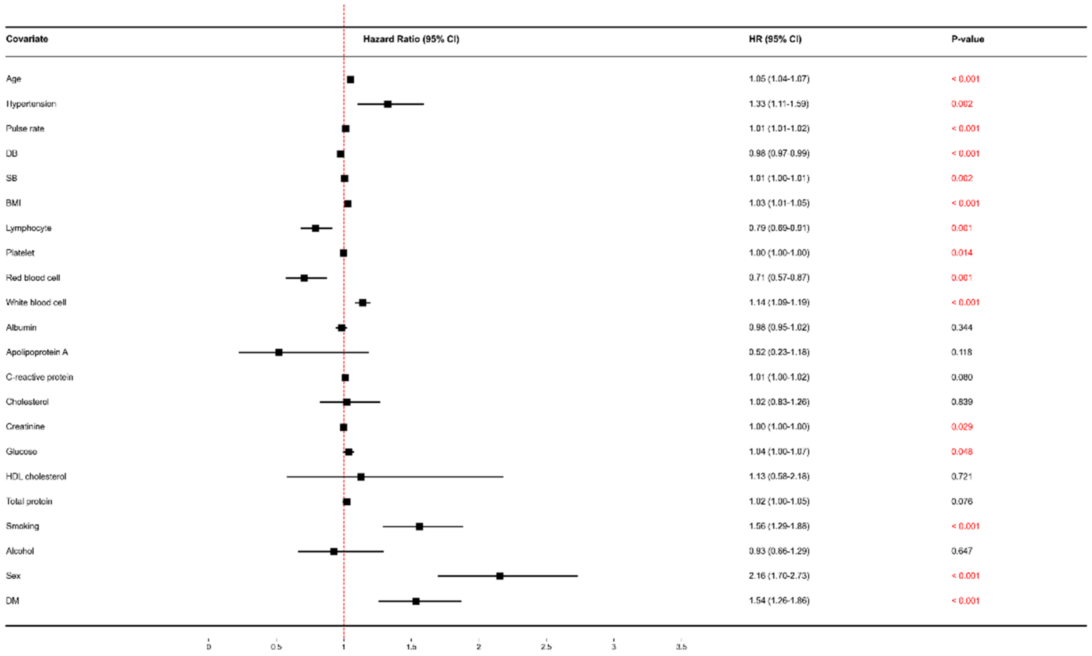

Figure S5. Forest plot of multivariable Cox regression analysis for acute myocardial infarction in patients with CHD.

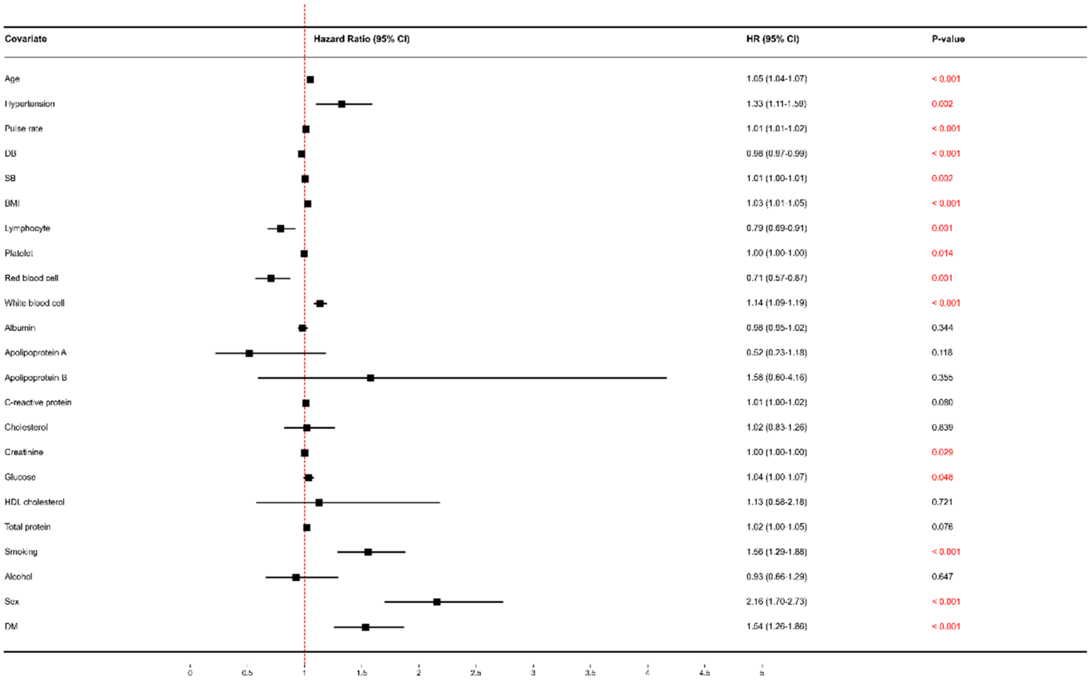

Figure S6. Forest plot of multivariable Cox regression analysis for stroke in patients with CHD.

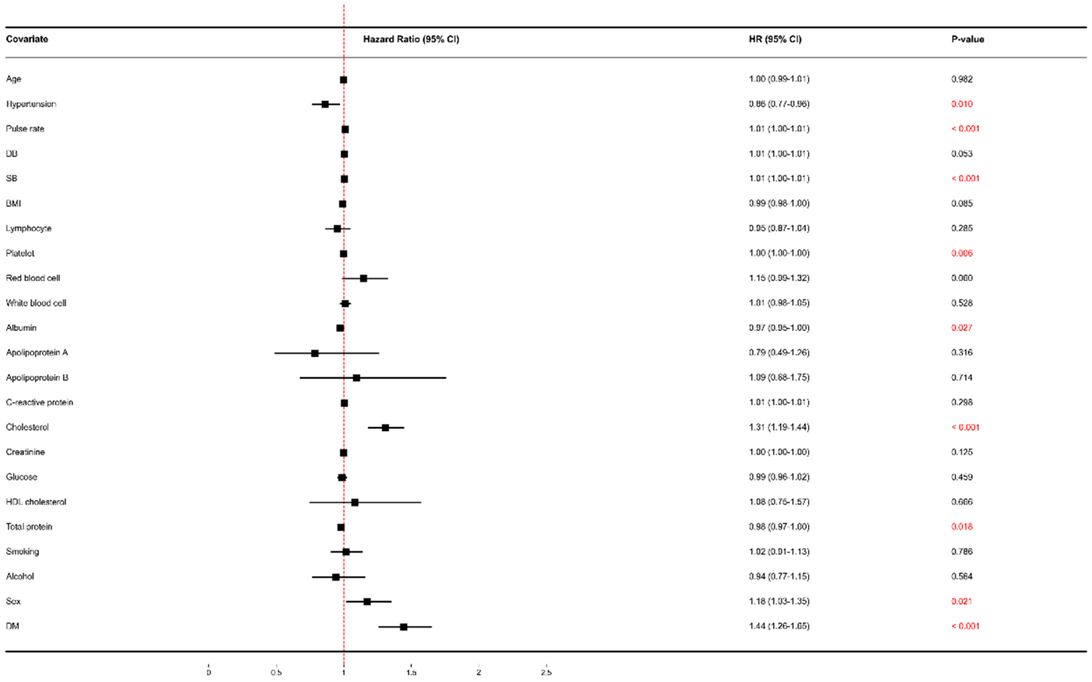

Figure S7. Forest plot of multivariable Cox regression analysis for MACE in patients of Tongji Hospital cohort.

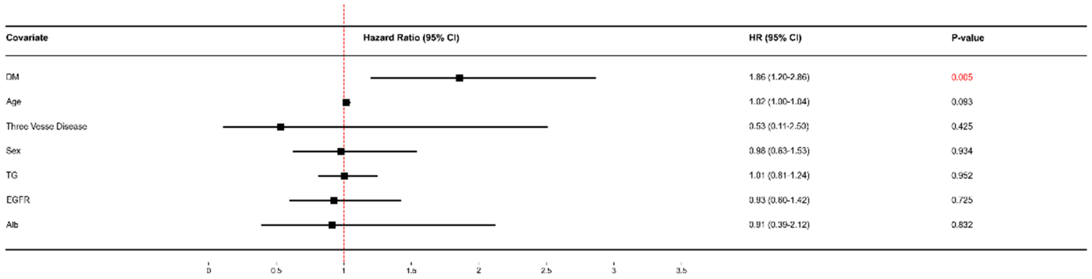

Supplement: Supplementary file 1 [file jcdd-13-00306-s001.zip › jcdd-4316706-supplementary.pdf]
